# Turmeric Herb Extract-Incorporated Biopolymer Dressings with Beneficial Antibacterial, Antioxidant and Anti-Inflammatory Properties for Wound Healing

**DOI:** 10.3390/polym15051090

**Published:** 2023-02-22

**Authors:** Piyachat Chuysinuan, Chalinan Pengsuk, Kriengsak Lirdprapamongkol, Thanyaluck Thanyacharoen, Supanna Techasakul, Jisnuson Svasti, Patcharakamon Nooeaid

**Affiliations:** 1 Laboratory of Organic Synthesis, Chulabhorn Research Institute, Bangkok 10210, Thailand; 2 Division of Biotechnology Technology and Agricultural Products, Faculty of Agricultural Product Innovation and Technology, Srinakharinwirot University, Ongkarak, Nakhon Nayok 26120, Thailand; 3 Laboratory of Biochemistry, Chulabhorn Research Institute, Bangkok 10210, Thailand; 4 Division of Polymer Materials Technology, Faculty of Agricultural Product Innovation and Technology, Srinakharinwirot University, Ongkarak, Nakhon Nayok 26120, Thailand

**Keywords:** wound dressing, antibacterial property, antioxidant activity, anti-inflammatory property, biopolymers, turmeric extract

## Abstract

Bacterial infection and inflammation caused by excess oxidative stress are serious challenges in chronic wound healing. The aim of this work is to investigate a wound dressing based on natural- and biowaste-derived biopolymers loaded with an herb extract that demonstrates antibacterial, antioxidant, and anti-inflammatory activities without using additional synthetic drugs. Turmeric extract-loaded carboxymethyl cellulose/silk sericin dressings were produced by esterification crosslinking with citric acid followed by freeze-drying to achieve an interconnected porous structure, sufficient mechanical properties, and hydrogel formation in situ in contact with an aqueous solution. The dressings exhibited inhibitory effects on the growth of bacterial strains that were related to the controlled release of the turmeric extract. The dressings provided antioxidant activity as a result of the radical scavenging effect on DPPH, ABTS, and FRAP radicals. To confirm their anti-inflammatory effects, the inhibition of nitric oxide production in activated RAW 264.7 macrophages was investigated. The findings suggested that the dressings could be a potential candidate for wound healing.

## 1. Introduction

Wound dressings are critical tools in the healing process of particularly chronic wounds arising from tissue injuries that heal slowly [1]. The characteristic properties of dressings play key roles in the treatment of chronic wounds. Dressings that are non-toxic and can protect wounds from contamination and further trauma and accelerate wound healing are needed [1,2]. Wound dressings must absorb excess exudates while maintaining moist environments at wound sites and have appropriate oxygen permeability [1,2,3]. Active dressings are the latest type of modern wound dressing produced based on biomaterials, which intrinsically show biocompatibility, biodegradability, and non-toxicity. Active dressings are usually incorporated with biological substances such as growth factors and drugs, and antibacterial agents to combat infections and enhance wound healing, especially chronic wounds [4]. Active dressings should have antioxidant properties to prevent excessive oxidation, to regulate inflammation, and hence to support wound repair [5,6]. The selection of dressing materials is essential for achieving wound repair in an orderly and timely manner. Biopolymers and synthetic polymers are used in fabricating currently available wound dressings in a variety of forms, such as films, hydrocolloids, fibers, foams, and hydrogels [2,7,8]. Hydrogels are potential dressing materials given that their interconnected porous structures mimic the physicochemical properties of tissue environments and absorb aqueous fluids [9]. Meanwhile, open-cell foams accumulate exudates and transmit moisture vapor and oxygen [1,9]. Highly porous dressings facilitate cell growth and subsequent tissue regeneration [5]. Porous materials with the ability to form gels in contact with fluids were thus of interest in the present study. Carboxymethyl cellulose (CMC) is one of the most extensively applied polysaccharide-based biopolymers used as a matrix in the fabrication of dressings because of its biocompatibility, biodegradability, and non-toxicity [2]. Given the high capacity for water absorption and good compatibility with the skin of CMC, it is physiologically harmless and inexpensive, and is a potential matrix in dressing materials [10,11]. In addition, it is hydrophilic, facilitates the formation of polysaccharide–protein complexes, and improves the mechanical properties of protein-based materials by blending with polysaccharides [12]. Silk sericin (SS) is a natural proteinous polymer known as a degumming waste product of silk cocoons commonly discarded in wastewater from textile plants [13,14]. Due to environmental pollution caused by the disposal of SS, a large volume of SS eliminated from silk manufacturing plants should be reused and converted into value-added products that contribute to sustainability. SS is composed of 18 types of amino acids and exhibits a hydrophilic nature, biocompatibility, and biodegradability [15,16]. Similar to silk fibroin, SS has been widely used as a promising sustainable biomaterial in various fields relevant to biomedical and pharmaceutical applications [17]. Especially in wound care, SS is an interesting and suitable material because of its moisture absorption, antioxidation, antityrosinase, and antibacterial activities [18]. Zhao et al. blended SS with chitosan and electrospun the materials to prepare porous wound dressings [16]. The results confirmed that the SS/chitosan dressings were compatible with fibroblasts and exhibited antibacterial properties [16]. SS-incorporated methacrylic-anhydride-modified gelatin hydrogel dressings promoted the adhesion of L929 cells and proliferation of HaCaT and HSF cells, establishing an appropriate environment for re-epithelialization; no inflammatory reaction was found, as reported in the study of Chen et al. [18]. SS promoted the proliferation of keratinocytes and fibroblasts, which are involved in cytokine production and re-epithelialization and in the production of extracellular matrix proteins for wound healing [18].

Since infections delay wound healing, the loading of medicinal herb extracts that have antibacterial, antioxidant, and anti-inflammatory properties in dressings has attracted considerable interest regarding the need to accelerate wound healing. *Curcuma longa* L. (turmeric), of the ginger family (*Zingiberaceae*), has been generally used as a safe and active drug to treat many chronic diseases. Turmeric contains several bioactive compounds, mainly including curcumin, demethoxycurcumin, bisdemethoxycurcumin, diterpenes, triterpenoids, and sterols [19]. Turmeric shows anti-inflammatory activity, a healing capacity, and antioxidant activity, and inhibits the growth of Gram-positive and Gram-negative bacteria, yeasts, and molds [20,21]. As a result, turmeric has been researched in various fields, including delivery systems, tissue engineering, and modern medicine [22,23,24].

To prevent bacterial infections without the use of antibiotics, and to simultaneously reduce oxidative stress and inflammation and support wound healing, turmeric extracts with different concentrations were loaded in dressings based on a combination of non-toxic, biocompatible, biodegradable, renewable, and cost-effective polysaccharide- and protein-based biopolymers. The physico-chemical, thermal, and mechanical properties, the release profiles, and the particular biological properties relevant to the regulation of wound healing of turmeric-incorporated CMC/SS dressings were investigated.

## 2. Materials and Methods

### 2.1. Extraction of Silk Sericin from Silkworm Cocoons

SS was extracted from Thai hybrid Bombyx mori silkworm cocoons (the local name is Leung Pairoj) with a slightly modified autoclave method [25]. Silkworm cocoons were collected after silk production and were supplied from Sakon Nakhon province in the northeastern region of Thailand. Prior to the extraction process, cocoons were cut into small pieces and washed with tap water twice for contaminant removal. Cocoon pieces were then placed in a drying oven at a temperature of 105 °C for 24 h. Dried cocoons were mixed with deionized water in a ratio of 1/30 (*w*/*v*). The mixture was placed in an autoclave (Tomy, SX-500, Tokyo, Japan) at a temperature of 121 °C for 30 min. The autoclaved mixture was filtered to obtain an SS solution. The solution was then frozen at −20 °C for 12 h and lyophilized in a freeze-drier at −50 °C under vacuum pressure for 48 h for the preparation of a dry SS powder.

Extraction of SS under pressure in an autoclave was successfully confirmed by using an attenuated total reflectance Fourier transform infrared spectroscopy (ATR–FTIR) system (Nicolet170-SX, Thermo Nicolet Ltd., Waltham, MA, USA). The chemical structure of the SS powder was analyzed over a wavenumber ranging from 4000 to 400 cm^−1^ at a resolution of 4 cm^−1^ for 64 scans at room temperature.

### 2.2. Fabrication of Turmeric-Loaded Carboxymethyl Cellulose/Silk Sericin Dressings

CMC solution at a concentration of 2% *w*/*v* was prepared by dissolving sodium CMC (Mw, 250,000; degree of substitution 0.7; Sigma-Aldrich, St. Louis, MO, USA) in deionized water and stirring it at room temperature. After the CMC solution was homogeneously obtained, SS powder was added to the CMC solution in a weight ratio of 1:1, and the mixture was continuously stirred until a homogeneous CMC/SS blended solution was obtained. For plant extract loading, turmeric (T; Code No. 4593-D420724, Thai-China Flavours and Fragrances Industry, Phra Nakhon Si Ayutthaya, Thailand) was added to the CMC/SS solution at concentrations of 1%, 2%, and 3% *w*/*v*. The CMC/SS and T-loaded CMC/SS solutions were crosslinked by an esterification reaction using 30% *w*/*w* citric acid (CA) 1-hydrate (analytical grade, Mw of 210.14 g/mol, Kemaus, New South Wales, Western Australia) in an aqueous solution at a temperature of 80 °C for 18 h. Finally, the crosslinked solutions were added to 24-well plates, frozen at a temperature of −20 °C, and lyophilized at −50 °C under vacuum pressure. Dried CMC/SS and T-CMC/SS dressings formed after 24 h of lyophilization.

### 2.3. Morphological Analysis

CMC/SS dressings with various concentrations (1%, 2%, and 3%T) were morphologically analyzed through scanning electron microscopy (SEM; JEOL, JSM-IT-500HR, Peabody, MA, USA) compared with pure CMC and SS dressings. Cylindrical samples were cut into small pieces and coated with gold by using a sputtering device prior to SEM observation. The pore sizes of the samples (300 randomly selected pores) were determined from SEM images by using ImageJ software.

### 2.4. Chemical Composition Analysis

The chemical composition of the CMC/SS and T-CMC/SS dressings was characterized using ATR–FTIR (Nicolet170-SX, Thermo Nicolet Ltd., Waltham, MA, USA). FTIR analysis was performed over a wavenumber range of 4000–400 cm^−1^ at a resolution of 4 cm^−1^ for 64 scans at room temperature.

### 2.5. Mechanical Test

The mechanical properties of the dressings were tested under compressive force. The diameter and thickness of cylindrical samples were determined prior to applying compressive force with a universal testing machine (Instron 5966, Norwood, MA, USA). The samples were compressed to 80% of their initial thickness at a crosshead speed of 2 mm/min and load cell of 50 kN. The mean values of compressive modulus and strength at 50% displacement were averaged from 10 specimens tested for each condition.

### 2.6. Thermal Transition Behavior and Stability Analysis

The thermal transitions of dressings were investigated by using a diffraction scanning calorimetry (DSC) system (NETZSCH DSC 204F1 Phoenix, Selb, Germany), with a heating rate of 10 °C/min and temperature ranging from 25 °C to 400 °C. The thermal stability of the dressings was analyzed by using a simultaneous thermal analysis (STA) system (NETZSCH5 STA 449F3, Selb, Germany). STA analysis was performed in a nitrogen atmosphere from 25 °C to 600 °C at a heating rate of 10 °C/min.

### 2.7. Water Absorption Study

The ability of the dressings to absorb water was investigated by immersion in the phosphate-buffered saline (PBS) solutions (pH 7.4; tablets; Mw 58.44 g/mol; Gold Biotechnology, St. Louis, MO, USA). Each cylindrical sample with a diameter of 14 mm and height of 5 mm was placed in a polystyrene bottle containing 50 mL of PBS solution. The samples were placed in an orbital shaker for 48 h. The orbital shaker was set at a temperature of 37 °C, and the shaking speed was set at 90 rpm. The sample was removed, excess water at the surface was blotted with filter paper, and the samples were weighed. Wet weight was recorded at each time (W_t_). At each time point, the water absorption of each type of sample was determined according to the initial dry weight (W_i_) with the following equation:

$$\mathrm{Water}\;\mathrm{absorption}\;\left(\%\right)=\frac{W_{t}-W_{i\;}}{W_{i}}\times 100$$

### 2.8. Determination of Encapsulation Efficiency (EE)

The capacities of CMC/SS dressings that encapsulated turmeric extract were determined by using an indirect quantification method. T-CMC/SS dressings were added to a sodium citrate solution at a concentration of 5% *w*/*v*. The mixtures were stirred at room temperature until the dressings were completely dissolved in the solution and separated through centrifugation at room temperature for 10 min. The supernatant was collected and we determined the amount of encapsulated turmeric extract at 425 nm by using a UV–Vis spectrophotometer (GENESYS 10S, Menlo Park, CA, USA). The EE was calculated with the following equation:

$$\mathrm{EE}\;\left(\%\right)=\frac{T_{e}}{T_{i}}\times 100$$

where T_e_ is the amount of turmeric extract encapsulated in the dressing and T_i_ is the initial amount of turmeric extract added to the dressing. The experiment was performed in triplicate.

### 2.9. Controlled Release and Kinetics Study

Cylindrical dressings with various concentrations of turmeric loading with a diameter of 14 mm and thickness of 5 mm were separately placed in a polystyrene bottle containing 50 mL of PBS solution as a releasing medium at pH 7.4. All experiments were carried out at 37 °C for 48 h with agitation at 90 rpm in an orbital shaker. At each immersion time, 1 mL of sample solution was withdrawn and used in determining the amount of turmeric released. Meanwhile, an equivalent amount of fresh PBS solution was replaced to maintain the sink condition. The amount of turmeric released at each time point was determined using the UV–Vis spectrophotometer at a wavelength of 425 nm. The absorbance of the detected extract was converted into an extract concentration according to the calibration curve of turmeric in a PBS solution, which was prepared by using a series of turmeric concentrations (0–50 mg/L). Results as % cumulative release as a function of immersion time were calculated using the following equation:

$$\mathrm{Cumulative}\;\mathrm{release}\;\left(\%\right)=\frac{M_{t}}{M_{0}}\times 100$$

where M_t_ is the cumulative release at time t and M_0_ is the initial concentration of a loaded extract. Four replicates were analyzed for each sample type, and results were presented as mean ± standard deviation (SD).

The release mechanism of T-CMC/SS dressings (1%, 2%, and 3%T loading) was investigated by fitting the turmeric release results to different drug release models, including the Higuchi and Korsmeyer–Peppas models [26,27]. The best fit was indicated by the determination of correlation coefficient (r) values. To understand the release kinetics, we fitted the release data of up to 60% cumulative release to mathematical models. The Higuchi equation is one of the most well-known controlled release kinetic equations involving the diffusion mechanisms of drugs released from drug delivery systems [26,28]. The Higuchi equation can be represented by the following equation:

$$M_{t}={\mathrm{kt}}^{0.5}$$

where M_t_ is the amount of released turmeric at time t, k is the Higuchi diffusion constant, and t is the investigation time. According to the Higuchi equation, the percent cumulative release at time t was plotted against the square root of time (t^0.5^).

The release data were fitted using the Korsmeyer–Peppas model, which is a well-known kinetic model for drug release from a polymeric system [26,28]. The dissolution mechanism is represented in the following equation:

$$\frac{M_{t}}{M_{\infty }}={\mathrm{kt}}^{n}$$

where M_t_/M_∞_ is the fraction of turmeric release at time t, k is the Korsmeyer–Peppas rate constant, and n is the release exponent or diffusional exponent. The n value indicates the release mechanism according to the calculated slope of the logarithm plot of M_t_/M_∞_ and time (t) [28,29]. In cylindrical samples, *n* < 0.45 indicated Fickian diffusion, 0.45 < *n* < 0.89 represented anomalous transport, and *n* > 0.89 indicated a case II transport mechanism (zero-order kinetic) [29,30].

### 2.10. In Vitro Antioxidant Characterization

To prepare a sample solution for antioxidant assays, the T-CMC/SS dressings with a diameter of 14 mm and thickness of 5 mm were immersed in 20 mL of methanol for 2 h in a water bath with shaking.

#### 2.10.1. DPPH Radical Scavenging Assay

The free radical activity of T-CMC/SS dressings was determined by using the 2,2-diphenyl-1-picrylhydrazyl (DPPH) radical scavenging assay, as previously described [31]. First, 1 mL of a sample solution was mixed with 3 mL of DPPH solution (0.1 mM in methanol). The mixture was incubated for 30 min in the dark. Finally, the absorbance of the reaction mixture was measured at 517 nm with a microplate reader. Radical scavenging activity (%) was calculated as follows.

$$\mathrm{DPPH}\;\mathrm{radical}\;\mathrm{scavenging}\;\mathrm{activity}\;\left(\%\right)=\frac{A_{\mathrm{Control}}-A_{\mathrm{Sample}}}{A_{\mathrm{Control}}}\times 100$$

#### 2.10.2. ABTS Radical Scavenging Activity

The ability of T-CMC/SS dressings to scavenge 2,2′-azinobis-(3-ethyl-benzothiazoline-6-sulfonate) (ABTS) radicals was determined by using the ABTS assay. First, an ABTS^+^ stock solution was prepared by mixing 2 mL of ABTS (7 mM) and 2 mL of potassium persulfate (4.95 mM), and the resulting solution was left in the dark at room temperature for 16 h. The ABTS^+^ working solution was prepared by diluting an ABTS^+^ stock solution with methanol, and the absorbance at 734 nm was 0.70 ± 0.02. The sample solution (100 μL) and ABTS^+^ working solution (200 μL) were mixed and incubated at room temperature for 2 h in the dark. The absorbance was measured at 734 nm, and the ABTS radical scavenging activity was calculated according to the following equation:

$$\mathrm{ABTS}\;\mathrm{radical}\;\mathrm{scavenging}\;\mathrm{activity}\;\left(\%\right)=\frac{A_{\mathrm{Control}}-A_{\mathrm{Sample}}}{A_{\mathrm{Control}}}\times 100$$

#### 2.10.3. Ferric Reducing Antioxidant Potential (FRAP) Assay

The antioxidant potential of T-CMC/SS dressings to reduce Fe^3+^ into Fe^2+^ was evaluated by using the slightly modified FRAP assay. A FRAP solution was prepared as a mixture of 33.3 mM ferric chloride solution: 9.9 mM tripyridyl-s-triazine: 300 mM acetate buffer (pH 3.6) in a 10:1:1 ratio. The sample solution (100 µL) was mixed in the FRAP solution (200 µL) and incubated at room temperature for 2 h. Then, the absorbance at 593 nm was measured in a microplate reader. The standard curve was generated using Trolox. FRAP antioxidant activity was expressed as milligrams of Fe^2+^ equivalent per gram of sample.

### 2.11. Antibacterial Test

The antibacterial activity of T-CMC/SS dressings was investigated against two pathogenic bacteria (Gram-positive and Gram-negative) by using the disk diffusion assay of the US Clinical and Laboratory Standards Institute, as previously described [32]. Bacterial suspensions (10^6^ CFU/mL) of *Staphylococcus aureus* (ATCC25923) and *Escherichia coli* (ATCC25922) were spread over agar plates and grown overnight at 37 °C. Dressings with a thickness of 2 mm were cut into circular disks (diameter of 14 mm). The sample disks were sterilized by exposure to UV light for 30 min prior to testing for bacterial inhibition by turmeric released from the T-CMC/SS dressings. CMC/SS samples without turmeric were used as controls. Each sample disk was placed on the bacterial agar plate and incubated at 37 °C for 24 h. Subsequently, the diameters of clear zones around the sample disks were measured. The normalized width of the antimicrobial halo (NW_halo_) of each sample disk was determined by applying the following equation according to [32]:

$${\mathrm{NW}}_{\mathrm{halo}}=\frac{\left(\frac{D_{\mathrm{iz}}-D}{2}\right)}{D}$$

where D_iz_ is the diameter of the inhibition zone (mm) after incubation for 24 h and D is the diameter of a sample disk (mm). Four replicates were tested for each sample.

### 2.12. In Vitro Cytotoxicity Test

An indirect cytotoxicity test was performed on T-CMC/SS dressings in accordance with the ISO10993-5 standard test method [6,29]. Briefly, the dressings (20 mg) were sterilized under UV radiation for 30 min and then immersed in 0.2 mL of phenol red-free DMEM cell culture medium (Gibco, Thermo Fisher Scientific, Waltham, MA, USA) supplemented with 10% *v*/*v* fetal bovine serum and 1% penicillin/streptomycin, in a 96-well tissue culture polystyrene plate (TCPS). They were incubated for 24 h to produce sample extracts for cytotoxicity and anti-inflammatory tests. RAW 264.7 cells (mouse macrophage cell line, ATCC TIB-71, Rockville, MD, USA) were seeded in a phenol red-free medium at 30,000 cells/100 µL/well in a separated 96-well TCPS and cultured at 37 °C for 24 h to allow cell attachment onto the well surface. Then, 25 µL of a sample extraction was added to each well, and the cells were further incubated for 24 h. Given that RAW 264.7 cells weakly attached to the plate, a modified MTT assay was used in determining the viability of the cells. After the treatment, the wells were mixed with an MTT solution (25 µL) and further incubated for 4 h. Then, 100 µL of lysis solution (20% SDS in 10 mM HCl) was added to lyse cells, and formazan crystals formed by the mitochondrial activity of living cells were solubilized. The plate was left in the dark at room temperature for 2 days. The absorbance at 550 nm was measured and subtracted from the absorbance at 650 nm. The absorbance of untreated control cells was collected upon 100% cell viability. Three replicates were investigated for each sample.

### 2.13. Anti-Inflammatory Assay

Anti-inflammatory effects of the T-CMC/SS dressings were determined according to their performance in inhibiting nitric oxide production in RAW 264.7 cells. A cell suspension (100 µL) in a phenol red-free medium was seeded into 96-well TCPS at 30,000 cells/well and cultured at 37 °C for 24 h. Then, the cells were treated by adding 25 µL of sample extract, incubated for 3 h, and stimulated with 10 µg/mL lipopolysaccharides (LPSs) from *E. coli* (Sigma Aldrich, Saint Louis, MO, USA) for 24 h. The culture medium in each well was collected and used in determining the nitric oxide level with a Griess reagent (Promega, Madison, WI, USA) [6]. Briefly, 50 µL of the collected medium was mixed with 100 µL of Griess reagent and incubated for 20 min at room temperature in the dark. The absorbance was measured at 540 nm, and the concentration of nitrite was calculated using a sodium nitrite standard curve (0–100 µM).

### 2.14. Statistical Analysis

A one-way ANOVA was used in comparing the means of different data sets, and *p* < 0.05 was considered statistically significant.

## 3. Results and Discussion

### 3.1. Microstructure

**Figure 1:**
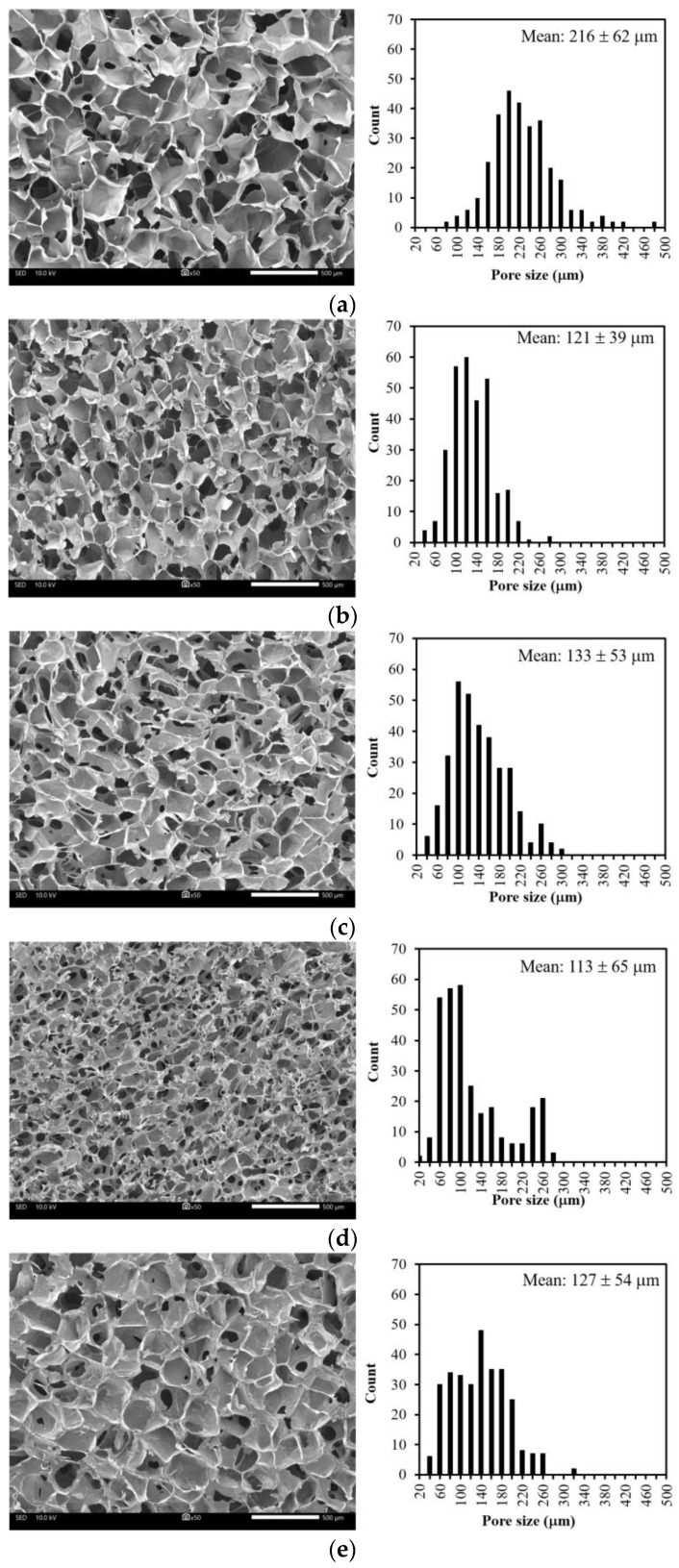
SEM micrographs showing cross-sectional morphology and pore size distributions of dressings including (**a**) CMC, (**b**) CMC/SS, (**c**) 1%T-CMC/SS, (**d**) 2%T-CMC/SS and (**e**) 3%T-CMC/SS. All of scale bars represent 500 μm.

CMC was successfully fabricated into three-dimensional (3D) porous dressings by chemical crosslinking with citric acid (CA). A highly interconnected porous structure showing a uniform opened cellular microstructure was observed, as shown in Figure 1a. The pore sizes of the CMC dressings ranged from 70 μm to 480 μm, and the mean pore size was determined at around 216 ± 62 μm. Figure 1b shows the maintained microstructures of the CMC dressings after blending CMC with SS. However, it was obvious that the pore sizes of the CMC/SS dressings were smaller than those of the CMC dressings, having a pore size distribution in the range of 30–280 μm after blending with SS. The mean pore sizes of the dressings decreased to 121 ± 39 μm in the presence of SS. It can be suggested that the reduced pore sizes of the CMC dressings with the presence of SS were probably due to the formation of a polysaccharide–protein complex [12,33]. For instance, hydrogen bonding between the -COOH group of the CMC polysaccharide and the -NH_2_ and -COOH groups of the SS protein led to the increased entanglement of biopolymer chains and consequently decreased their free volume. After the incorporation of turmeric extract at different concentrations, changes in morphology and pore dimension were not significant. Figure 1c–e show that the turmeric-loaded dressings had highly porous microstructures with the same pore size (approximately 20–300 μm) as the CMC/SS dressings without turmeric extract. The result suggests that the turmeric loading did not intensively impact the microstructure of the porous CMC/SS dressings. Moreover, an increase in the turmeric amount did not significantly alter the pore sizes of the dressings. A highly interconnected porous structure with opened pores was maintained, which is suitable for absorbing large amounts of exudates, facilitating cell migration and oxygen and other nutrients’ transfer, thus improving cell growth and tissue regeneration [17]. In addition, the pore sizes of the T-CMC/SS dressings (30–300 μm) were in the same range as those of commercially available foam dressings (around 32–1000 μm) [34].

### 3.2. Chemical Compositions

**Figure 2:**
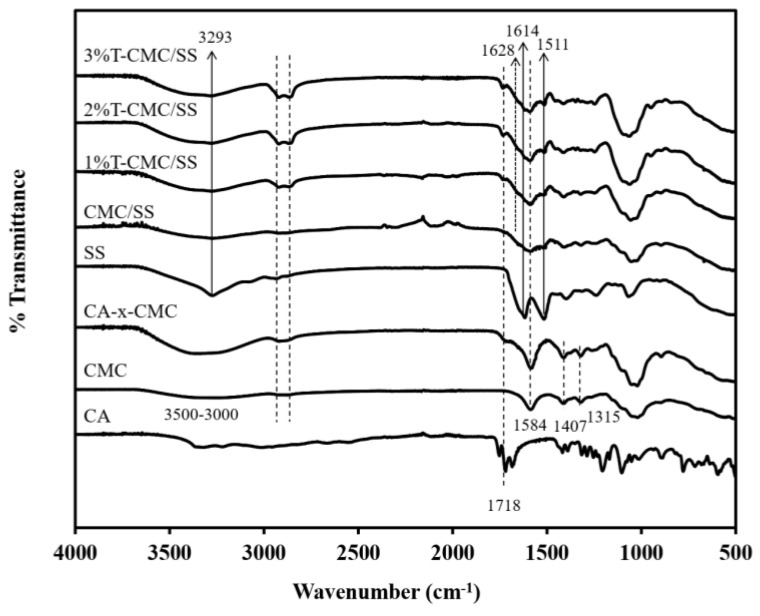
FTIR spectra of dressings including neat citric acid (CA), neat CMC (CMC), as-extracted SS, and citric acid-crosslinked dressings composed of citric acid-crosslinked CMC (CA-x-CMC), crosslinked CMC/SS (CMC/SS), and crosslinked CMC/SS with loading of turmeric extract (T-CMC/SS).

Figure 2 shows the FTIR spectra of fabricated CMC/SS dressings with and without turmeric loading compared with the spectra of the neat materials used. In the study, the stability of the dressings in an aqueous environment was enhanced by crosslinking CMC-based dressings with citric acid (CA), given that CMC is a hydrophilic polymer. CA is a naturally occurring carboxylic acid and was used as a crosslinking agent for CMC through an esterification reaction. The spectrum of CMC without crosslinking showed strong absorption peaks at 1584 cm^−1^ and 1407 cm^−1^, assigned to the asymmetric and symmetric stretching of carboxylate (COO^−^) groups, respectively. The peak at 1315 cm^−1^ corresponded to CH-O-CH_2_ stretching. A broad absorption band at a range of 3600–3000 cm^−1^ was due to –OH stretching [35]. After crosslinking with CA, an additional peak was found at a wavenumber of 1718 cm^−1^ in the spectrum of CA-x-CMC that was attributed to the formation of ester linkages during the crosslinking reaction. The presence of an ester bond confirms the occurrence of crosslinking. The peak intensity of the –OH stretching vibration of CMC at 3600–3000 cm^−1^ obviously increased after crosslinking, indicating that intramolecular and intermolecular interactions formed during esterification. The absorption peak at 1584 cm^−1^ in the spectrum of CA-x-CMC was stronger than that of CMC because of the formation of the carboxylate anion (COO^−^ group) during crosslinking [35,36]. The findings confirm that the crosslinking reaction between CMC and CA was successfully achieved, when compared with the spectra of neat CMC and CA. In the FTIR spectrum of SS, characteristic peaks of SS were found at 3293 cm^−1^, which was associated with N-H stretching vibration and overlapped with the band of –OH stretching at 3600–3000 cm^−1^. In addition, C=O in the amide group and N-H bending were found at 1614 and 1511 cm^−1^, respectively [15]. This means that the autoclave method is an effective and eco-friendly method for extracting SS from silk cocoons. The spectrum of CMC/SS dressings showed the characteristic peaks of CMC and SS. The peak at a wavenumber of 1584 cm^−1^ was due to the stretching vibration of C=O in CMC, whereas the vibrations of N-H stretching, C=O stretching of amide groups, and N-H bending in the chemical structure of SS were found at 3293 cm^−1^, 1614, cm^−1^, and 1511 cm^−1^, respectively. The intensity of the broad band at 3600–3000 cm^−1^ showed that the free hydroxyl groups decreased relative to that of the CMC dressings. This decrease indicates intermolecular interactions between polysaccharides and proteins, such as hydrogen bonding between CMC and SS [37]. The molecular interaction between CMC and SS contributed to the stability of the porous CMC/SS dressings [17]. The finding also relates to the reduction in the CMC dressings’ pore sizes with SS blending. The FTIR spectra of the T-CMC/SS dressings were observed in the same peak positions as the spectrum of the CMC/SS dressings, but a peak appeared at 1628 cm^−1^, which is a characteristic position of aromatic moiety C=C stretching in turmeric extract [20]. It can be suggested to be due to the existence of the turmeric extract loaded in the dressings.

### 3.3. Thermal Properties

**Figure 3:**
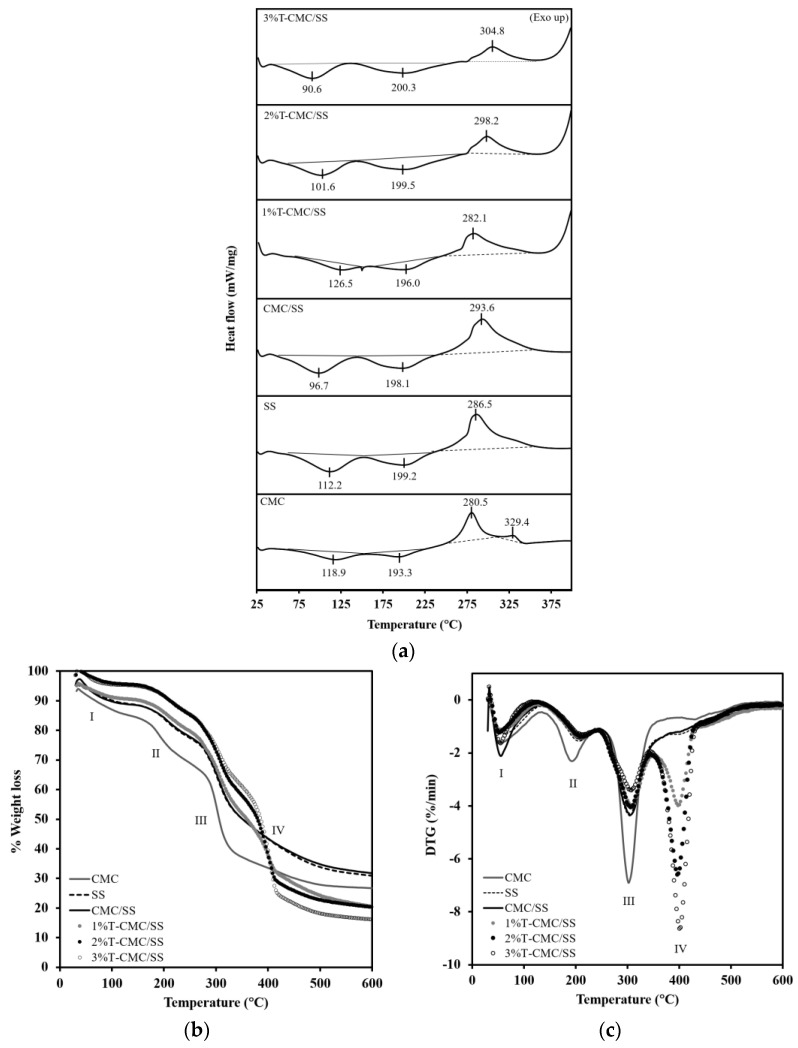
(**a**) DSC patterns, (**b**) TGA curves, and (**c**) DTG curves of CMC, SS, CMC/SS, and T-CMC/SS dressings.

DSC analysis was used in investigating the thermal stability of the T-CMC/SS dressings compared with that of the CMC and SS dressings. As illustrated in Figure 3a, all sample types showed an endothermic transition in a temperature range of 50–150 °C, as a typical result of water evaporation in hydrophilic polymers. Another endothermic peak was found at around 150–225 °C that was attributed to the thermal decomposition of the CMC and SS biopolymers. In addition, an obvious exothermic transition was observed at around 250–375 °C in all dressings and was attributed to the degradation temperature (T_d_) of the materials. Blending CMC with SS enhanced the thermal stability of CMC. The T_d_ of CMC/SS dressings appeared at 293.6 °C, whereas the T_d_ of the CMC and SS dressings were found at 286.5 and 280.5 °C, respectively. After the turmeric extract was loaded, a change in T_d_ was not observed, indicating that the presence of the turmeric extract in the CMC/SS dressings did not influence the thermal transition behavior of the dressings. The thermal stability of dressings maintained under thermal conditions during sterilization was confirmed by TGA and DTG results. In Figure 3b, the thermogram of the CMC dressings shows three regions of weight loss, including (I) the evaporation of free water and moisture trapped inside the porous structures, ranging from 40 °C to 125 °C; (II) the occurrence of the evaporation of water bound in the cellulosic structure and the decomposition of crosslinked CA at around 135–245 °C [36,38]; and (III) a large region showing the pyrolytic decomposition of the polymer backbone (T_d_) at 245–365 °C. At this stage, the mass loss of the polymer was found at around 42.75%. This result agrees with the DTG curves in Figure 3c. Three major endothermic peaks appeared in the CMC dressings’ curve. SS dressings showed a TGA curve similar to the CMC dressings’ curve, composed of three main decomposition stages. The desorption of bound water was observed up to 120 °C. The thermal decomposition regions of the SS component were found at 160–270 °C and 250–305 °C, with 11.37% and 46.68% weight loss, respectively. In the curve of the CMC/SS dressings, the addition of SS slightly increased T_d_ to 306.1 °C, compared with 302.9 °C in CMC (Figure 3c). SS blending can slow down the thermal decomposition of dressings, in contrast to the use of individual polymers. In the T-CMC/SS dressings, the TGA curves exhibited an additional decomposition step (IV) in a temperature range of 350–450 °C, involving the decomposition of the incorporated turmeric extract. The DTG curves in Figure 3c agree with the TGA curves. T-CMC/SS dressings revealed four dominant peaks. The maximum decomposition temperatures of turmeric loaded in CMC/SS dressings at concentrations of 1%, 2%, and 3% were found at 398.5 °C, 397.0 °C, and 401.3 °C, respectively. This finding means that the step height of the TGA curves and the peak area of the DTG curves increased with the concentration of turmeric loading. Moreover, the degradation temperature of turmeric incorporated into a polymer matrix was higher than that of pure turmeric extract (280 °C). The probable reason was the interaction of extract molecules with CMC and SS biopolymers through hydrogen bonding, protecting the turmeric compound from thermal degradation better than a free compound. This phenomenon was also found in a previous study [39]. According to DSC, TGA, and DTG thermograms, the CMC/SS dressings could be used as a protective carrier of turmeric extract to maintain its bioactivity under thermal conditions, especially during the sterilization process (i.e., 121 °C by using autoclave method).

### 3.4. Mechanical Properties

**Figure 4:**
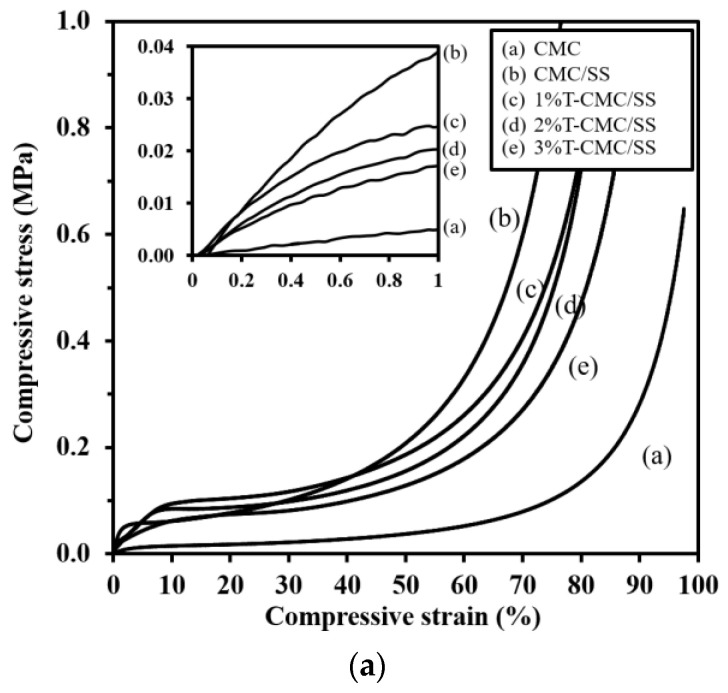

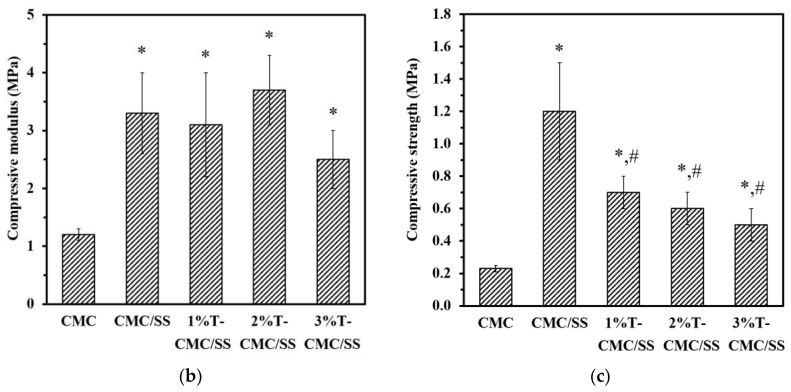
(**a**) Representative compressive stress–strain curves of dressings (the inlet shows the stress–strain curves in an elastic region, indicating the compressive modulus of the dressings), (**b**) compressive modulus, and (**c**) compressive strength of the dressings. Significant differences when compared to pure CMC dressings are indicated by * (*p* < 0.05), and significant differences when compared to CMC/SS dressings are indicated by # (*p* < 0.05).

Biopolymers typically have low mechanical properties compared with synthetic polymers, and this property is a limitation in various applications. In this study, SS with intrinsic stiffness was selected for blending with CMC in order to enhance the mechanical performance of the dressings. Figure 4a shows the compressive stress–strain curves of representative dressings. All types of samples exhibited the same mechanical behavior under compressive force, which is a typical curve of porous polymeric materials. The slopes of curves in the elastic region were calculated to determine the materials’ stiffness (an inset in Figure 4a). The mechanical properties of the CMC dressings in terms of compressive modulus and strength significantly improved after blending with SS. As shown in Figure 4b,c, CMC/SS dressings had the compressive modulus and strength of 3.3 ± 0.7 MPa and 1.2 ± 0.3 MPa, respectively, whereas the modulus and strength of pure CMC dressings were 1.2 ± 0.1 and 0.23 ± 0.02 MPa, respectively. The SS plays a key role in enhancing the elastic modulus and mechanical strength of CMC dressings by functioning as a reinforcing additive. The improvement in the mechanical properties found in the CMC/SS dressings was in agreement with the formation of the polysaccharide–protein complex. The incorporation of turmeric extract did not remarkably influence the compressive modulus of the CMC/SS dressings (Figure 4b). Meanwhile, an increase in turmeric content significantly reduced the compressive strength of the dressings from 1.2 ± 0.3 MPa to 0.5–0.8 MPa, as presented in Figure 4c. It can be suggested that the turmeric compound in the dressings could act as a plasticizer in the polymer network, leading to an improvement in the dressings’ softness. Softness and flexibility are characteristics of the ideal dressing as it can be removed without causing pain and trauma to the wound. In addition, the mechanical and structural stability of the dressings are substantiated in comfortable clinical trials and they are capable of frequent changes [40].

### 3.5. Water Absorption Behaviors

**Figure 5:**
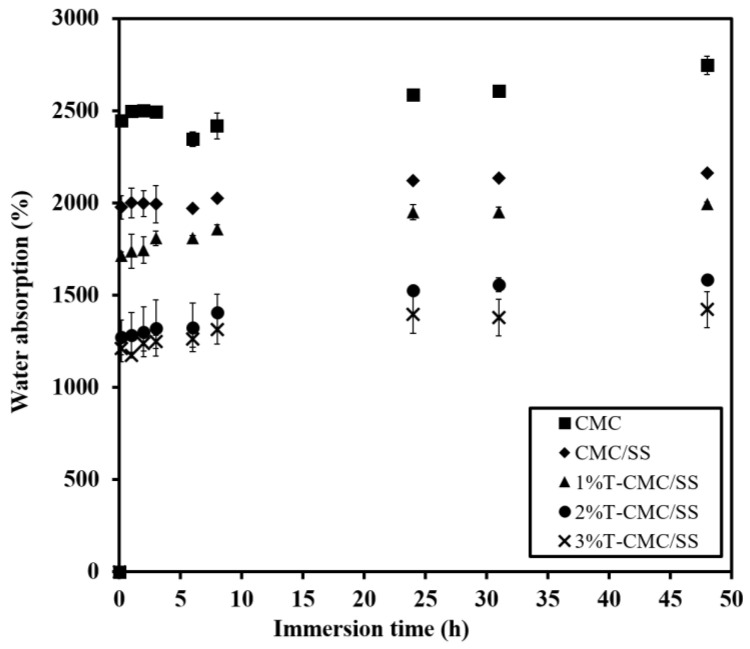
Water absorption of CMC/SS and T-CMC/SS dressings investigated by immersion in PBS solution (pH 7.4, 37 °C) for 48 h.

After the dressings were immersed in PBS solutions, all types of samples showed fast water uptake at an initial time of 10 min and subsequently formed hydrogels by the first hour of immersion, as shown in Figure 5. Pure CMC dressings gained the highest amount of water at around 2500%, and the addition of SS, forming CMC/SS dressings, led to a slight reduction in water uptake (~2000%). As a result of turmeric extract loading, T-CMC/SS dressings exhibited reduced water absorption to around 1000–1750%. The finding agrees with the dressings’ porous microstructure, as previously mentioned. It was also found that the fluid absorption capacity was 10–20 times the sample weight, while their structural stability was maintained at an equilibrium point for 48 h of investigation without disintegration. The result showed the same range as the absorption ability of commercially available alginate dressings (15–20 times), as previously reported [40], which indicates that these are suitable dressings for highly exuding wounds. The high water absorption ability of dressings has been reported to aid granulation tissue in a moist environment [5]. The formation of hydrogels also promoted a soothing effect by reducing the temperature of the wound. Hence, wetted dressings increased the healing rate in contrast to dry dressings, because the healing of wounds without inflammation takes place in a moist environment [1].

### 3.6. Release Behaviors and Kinetics

**Figure 6:**
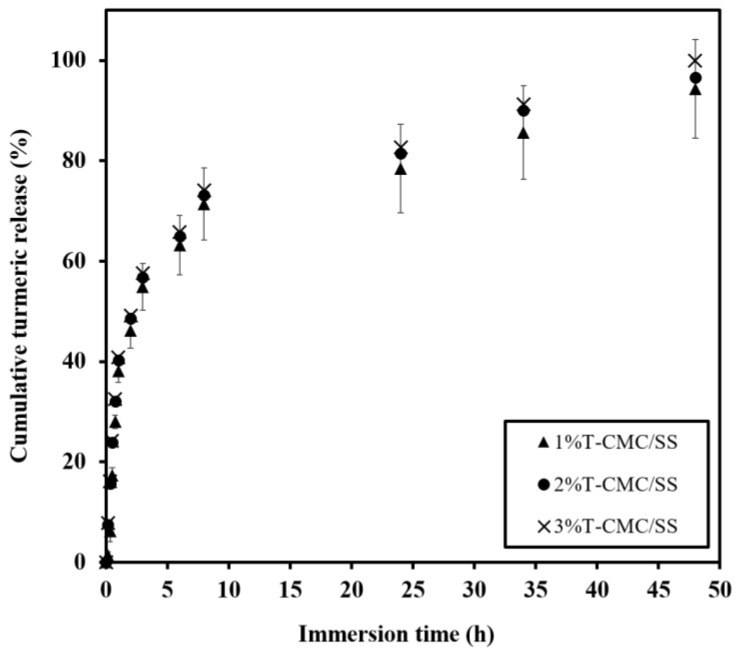
Release profiles of turmeric extract released from T-CMC/SS dressings investigated by immersion in PBS solutions (pH 7.4 at 37 °C) for 48 h.

CMC/SS dressings were expected to serve as an active delivery device that enables the controlled release of bioactive compounds such as loaded turmeric extract and to prevent the risk of an overdose effect of antibiotics. CMC/SS dressings loaded with turmeric extract at 1%, 2%, and 3% *w*/*v* showed encapsulation efficiency of 80 ± 8%, 85 ± 5%, and 96.8 ± 0.7%, respectively. It is suggested that the highly porous CMC/SS dressings were able to encapsulate high amounts of turmeric extract, indicating a potential carrier of hydrophilic compounds. Such high loading capacity was due to the good compatibility between the hydrophilic biopolymer matrix and water-soluble turmeric extract. In other words, the turmeric compound was well embedded in the network of crosslinked biopolymers. Figure 6 shows the similarity of the release behaviors among 1%, 2%, and 3%T-CMC/SS dressings. By immersion in a PBS solution for 48 h, each type of dressing exhibited a typical burst release up to 40% in the initial first hour of investigation, followed by the gradual increase in turmeric release at a long immersion time. It was noticed that the initial burst release matched the high water uptake rate (Figure 5). The high diffusion capacity of the dressings might induce the burst release of the turmeric extract. Moreover, it was due to the fast release of the turmeric compound weakly bound at the dressings’ surfaces. After 6 h of immersion, the dressings reached an effective level in a therapeutic concentration range of 60–80% until soaked for 24 h. For an extended time, the turmeric extract released from all dressings slightly increased, followed by sustained release over 48 h. At 48 h of immersion, turmeric release was observed at 90–100% in all cases. An increased amount of turmeric loading raised the released turmeric concentration in the medium at all time points. The release behavior of turmeric extract from CMC/SS dressings was found in an equivalent manner to that reported in a previous study of guava leaf extract-loaded alginate/gelatin hydrogels, which displayed an initial fast release within 30 min, followed by gradual release after 1 h of immersion in a PBS solution [31]. In addition, the release study of curcumin-loaded polycaprolactone dressings demonstrated controlled release behavior over 48 h of investigation [41].

To investigate the release mechanism of turmeric extract incorporated in CMC/SS dressings, release kinetics models such as the Higuchi and Korsmeyer–Peppas models were applied to find the best fit with the turmeric release behavior (Figure S1). Release parameters, such as the release constant (k) and release exponent (n), were determined and used in identifying the release mechanism, whereas the r^2^ coefficient of determination over 0.95 was considered to indicate the goodness of fit of the release model [42]. As seen in Table 1, the turmeric extract released from all formulations showed the best fit for the Higuchi and Korsmeyer–Peppas models, as indicated by the r^2^ values higher than 0.97%. The extract released from the dressings followed the Higuchi model, which is typically used to describe the controlled release of water-soluble molecules based on a Fickian diffusion mechanism [43,44]. The turmeric extract (1%, 2%, and 3%T) loaded in the dressings did not present a significant difference in Higuchi constant (k), ranging from 0.56 to 0.64, indicating that the diffusion rate of all formulations was not influenced by the turmeric loading content. According to the Korsmeyer–Peppas model, the release exponent (n) values can be used in characterizing the release mechanisms of polymeric materials. The dressings with different 1%, 2%, and 3%T loading had n values of 1.89, 0.94, and 0.93, respectively. The n values above 0.89 indicated that the release of the turmeric compound from CMC/SS dressings was controlled by the mechanism of case II transport, which refers to first-order kinetics, indicating that the release is controlled by the swelling of the matrix [30]. Thus, it can be suggested that the release behavior of T-CMC/SS dressings was driven by the diffusion mechanism and swelling capacity. This finding was supported by the high water absorption capability as the dressings tended to gain high water content and consequently became swollen hydrogels at the initial period of immersion in PBS solution. The obtained data supported the hypothesis that the T-CMC/SS dressings with the in situ formation of a hydrogel in contact with aqueous conditions would be appropriate for healing highly exudating wounds, and they could prevent the wound from dehydration. Subsequently, such moist environments induced the release behavior of the turmeric compound through the diffusion and swelling mechanism of the dressings. The ability of the dressing to provide a predictable and sustainable release rate plays a key role in the antibacterial function to combat infections.

### 3.7. Antibacterial Activity

Infection caused by bacterial contamination is known as a fundamental problem in chronic wounds, since it prolongs the inflammatory phase of wound healing, leading to a delay in wound repair [45]. For optimal wound repair, the elimination of bacterial contamination is necessary in the wound environment. Various antibiotics and synthetic antibacterial agents such as silver nanoparticles (AgNPs) have been widely researched for this purpose. For instance, riclin-AgNPs-based hydrogels as antibacterial and anti-inflammatory wound dressings were presented [46]. Even though AgNPs provided a broad antibacterial spectrum against both Gram-positive and Gram-negative bacterial strains, individual use of AgNPs was not responsible for the anti-inflammatory function. The AgNPs were therefore combined with ruclin to promote inflammation regulation. In addition, common side effects and the overusage of antibiotics become a severe problem for health. Thus, plant-based compounds and herbal medicinal extracts are recommended by the World Health Organization (WHO) as a safe and effective alternative to synthetic drugs [45]. In the present study, turmeric, extracted from a typical medicinal herb, was used as an antibacterial agent and as an antioxidant and anti-inflammatory agent.

The performance of the loaded turmeric extract in the CMC/SS dressings, inhibiting the growth of tested bacteria, is reported in Table 2, in comparison with that of CMC and CMC/SS dressings as controls. The bacterial growth inhibition activity of the dressings was determined by the halo diameter around the disk-shaped sample (Figure S2). The growth-inhibitory effect of the T-CMC/SS dressings was markedly observed in both bacterial strains *S. aureus* and *E. coli*. The NW_halo_ values of T-CMC/SS, ranging from 0.24 to 0.39, were correlated with the % turmeric loading of the dressings, in a concentration-dependent manner. The CMC and CMC/SS dressings did not exhibit antibacterial activity. Turmeric extract released from the dressings in the therapeutic concentration range of 60–80% within 24 h of immersion, as previously reported in Figure 6, was responsible for the antibacterial function of the dressings. The dressings with 1%–3%T loading exhibited potent antibacterial property, which is a desirable characteristic in active dressings to protect wounds from infection with pathogen microorganisms and to achieve effective wound healing.

### 3.8. Antioxidant Activity

A complicated healing process occurs via the excessive production of free radicals in response to tissue damage, since the free radicals destroy proteins, lipids, and the extracellular matrix (ECM) [46]. Apart from antibacterial properties, the fabricated active dressings were required to provide an antioxidant function to reduce free radicals and support fast wound healing. As a result, turmeric extract was selected, since it is an effective natural compound having various biological properties. In the current study, three different antioxidant assays, namely the DPPH, ABTS, and FRAP assays, were conducted to assess the in vitro antioxidant potential of T-CMC/SS dressings. The DPPH and ABTS free radical scavenging assays are based on the quenching of stable colored free radicals, indicating the potential scavenging ability of the test samples. As shown in Table 3, the DPPH and ABTS free radical scavenging activities of T-CMC/SS dressings were parallelly increased with the % turmeric loading, and the highest DPPH and ABTS free radical scavenging activities obtained by 3%T-CMC/SS dressings were 13 ± 2% and 24 ± 1%, respectively. To support the results of the DPPH and ABTS assays, a FRAP assay was also carried out. Similarly, the T-CMC/SS dressings displayed FRAP activity in a concentration-dependent manner, and the highest FRAP activity of 3.7 ± 0.4 mg Fe^2+^ equivalents/mg sample was obtained by the 3%T-CMC/SS dressings, contrary to the pristine dressings, which exhibited negligible radical scavenging capacity. The results are consistent with the report by Fan Y. et al. [47], as the DPPH radical scavenging property of turmeric extract increased with increasing concentrations. The effective antioxidant characteristics of turmeric extract were due to the presence of hydroxyl moieties, double bonds, and carbonyl groups [20]. It showed that the fabricated T-CMC/SS dressings could serve as an active antioxidant dressing by using a natural turmeric extract. This is a vital function in controlling the normal physiology of wound healing by maintaining low levels of wound oxidative stress [47].

### 3.9. In Vitro Cytotoxicity and Anti-Inflammatory Activity

**Figure 7:**
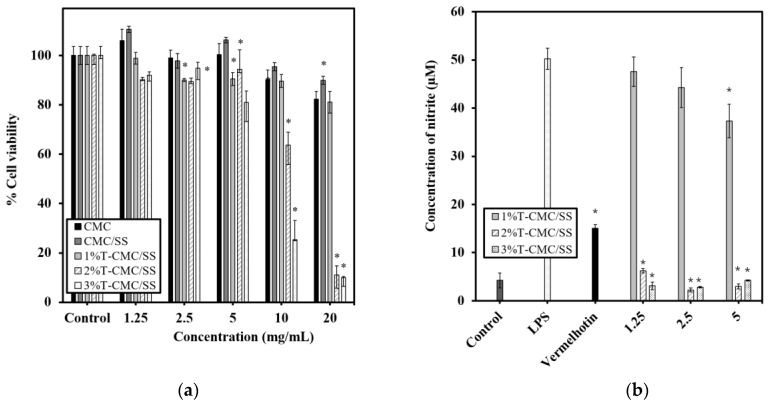
Cytotoxicity and anti-inflammatory effects of scaffold extracts in RAW 264.7 macrophages. (**a**) Viability of cells after exposure to sample extracts of 1%T-, 2%T-, and 3%T-CMC/SS dressings for 24 h. Cells cultured without sample extracts were used as control. Significant difference from untreated control cells is shown by * (*p* < 0.05). (**b**) Nitric oxide production in activated RAW 264.7 cells is indicated by the concentration of nitrite. The LPS-stimulated cells were treated with the sample extracts of 1%T-, 2%T-, and 3%T-CMC/SS dressings at selected non-cytotoxic concentrations of 1.25, 2.5, and 5 mg/mL. Vermelhotin (10 µM) was used as a positive control inhibitor. Significant difference from LPS-stimulated cells is indicated by * (*p* < 0.05).

RAW 264.7 macrophage-like cells were treated with the sample extracts of CMC, CMC/SS, and 1%, 2%, and 3%T-CMC/SS dressings at various concentrations (1.25, 2.5, 5, 10, and 20 mg/mL) for 24 h, and the cytotoxicity of the sample extracts was evaluated by using the MTT assay. The results showed that both CMC and CMC/SS dressings were not toxic to the cells at all tested concentrations, as the viability of the treated cells was greater than 80% (Figure 7a). The polymer matrices used in fabricating the dressings are not toxic and are biocompatible with the tested cells. In the presence of turmeric extract, the sample extracts of 2%T- and 3%T-CMC/SS dressings at concentrations of 10 and 20 mg/mL exhibited significant cytotoxic effects (*p* < 0.05), indicating an overdose of turmeric to the cells. Therefore, the concentration range of 1.25–5 mg/mL was considered a non-toxic dose for the sample extracts of 1%–3%T-CMC/SS dressings and was further used in evaluating the anti-inflammatory effect of the dressings in LPS-stimulated RAW 264.7 cells.

Nitric oxide (NO), produced by cells, is an inflammatory mediator that is crucial for wound repair. However, excessive NO production causes the dysfunction of tissue cells [47,48,49]. Therefore, the regulation of NO production is another significant role of suitable active dressings for wound healing. LPS stimulation markedly increased the nitric oxide production in RAW 264.7 cells to a level of 50 ± 2 µM, compared with 4 ± 2 µM in untreated control cells (Figure 7b). The sample extracts of 1%T-CMC/SS dressings decreased LPS-induced nitric oxide production, in a dose-dependent manner, and 37 ± 3 µM was observed at the highest tested concentration (5 mg/mL). Interestingly, the sample extractions of 2%T- and 3%T-CMC/SS dressings, at all tested concentrations, significantly reduced nitric oxide production to 2–6 µM, which is lower than 15.1 ± 0.7 µM observed in vermelhotin-treated cells. The results demonstrated that the anti-inflammatory effects of 2% and 3%T-CMC/SS dressings were greater than those of 1%T-CMC/SS dressings. Meanwhile, there was no significant difference in the effects of 2%T- and 3%T-CMC/SS dressings. It could be suggested that the turmeric content at 2% *w*/*v* was the optimal loading for the CMC/SS dressings to express effective anti-inflammatory properties. The 2%T-CMC/SS dressings are recommended as optimal active dressings with multifunctional features (antibacterial, antioxidant, and anti-inflammatory properties) for wound healing applications.

## 4. Conclusions

T-CMC/SS dressings were fabricated based on the use of biodegradable and renewable biopolymers through a green, facile, economic methodology. The T-CMC/SS dressings showed highly interconnected porous structures having pore sizes of around 30–300 μm with uniformity. The mechanical properties and thermal stability of the dressings were improved by blending CMC polysaccharide and SS protein. The dressings could be effective carriers of turmeric extract, as shown by the higher than 80% encapsulation efficiency. The dressings provided excellent water absorption capability for 10–20 times based on the weight of the dressings, which are appropriate to absorb high levels of wound exudate. Simultaneously, the dressings were able to form a hydrogel structure in contact with an aqueous solution, leading to the formation of moist dressings. Turmeric extract was released from the CMC/SS dressings in a controlled release manner over 48 h of investigation, including an initial burst release of up to 40% after the first hour, followed by gradual release at up to 60–80% after 24 h of investigation. The turmeric release behaviors were best fitted with the Higuchi and Korsmeyer–Peppas models. This evidenced that the diffusion rates of T-CMC/SS dressings were not influenced by the turmeric loading concentration, and the release behavior of turmeric extract from the dressings was controlled by the diffusion mechanism and the swelling of the dressings. The antioxidant activity of the dressings presented a concentration-dependent manner, given that the highest antioxidant activity was found in the case of 3%T-CMC/SS dressings. In addition, the 3%T-CMC/SS dressings exhibited the highest antibacterial activity against *E. coli* and *S. aureus* bacterial strains within 24 h of incubation, compared to other dressing types. The in vitro cytotoxicity test showed that the biopolymer matrices and the turmeric extract used for the fabrication of dressings were not toxic to RAW 264.7 macrophages. Importantly, the turmeric extract released from T-CMC/SS dressings was able to inhibit nitric oxide production by the activated RAW 264.7 cells, indicating anti-inflammatory properties. In particular, 2%–3%T-CMC/SS dressings exhibited highly efficient antibacterial, antioxidant, and anti-inflammatory activities. In combination with the specific physical properties of the T-CMC/SS dressings, including the highly porous microstructure, good thermal stability, enhanced mechanical properties, and high water absorption capability, it is recommended that the proposed dressings (the optimal 2%T-CMC/SS formulation) could be a potent candidate for use as bioactive dressings suitable for wound healing purposes. Significantly, turmeric extract could act as a multifunctional bioactive agent that is beneficial for would healing.

## Figures and Tables

**Table 1:** Modeling results of turmeric extract released from T-CMC/SS dressings fitted with Higuchi and Korsmeyer–Peppas release kinetics models.

Dressing	Release Kinetics Models			
	Higuchi		Korsmeyer–Peppas	
	k	r^2^	n	r^2^
1%T-CMC/SS	0.64	0.9772	1.89	0.9776
2%T-CMC/SS	0.56	0.9983	0.94	0.9929
3%T-CMC/SS	0.56	0.9985	0.93	0.9935

**Table 2:** Antibacterial activity of T-CMC/SS dressings at different concentrations of 1%, 2%, and 3% *w*/*v* against Gram-positive bacteria *Staphylococcus aureus* (*S. aureus*) and Gram-negative bacteria *Escherichia coli* (*E. coli*), determined by disk diffusion assay.

Dressing	Normalized Width of Antimicrobial Halo (NW_halo_)	
	*S. aureus*	*E. coli*
CMC	-	-
CMC/SS	-	-
1%T-CMC/SS	0.30 ± 0.04	0.24 ± 0.04
2%T-CMC/SS	0.33 ± 0.03	0.33 ± 0.02
3%T-CMC/SS	0.39 ± 0.03	0.37 ± 0.05

**Table 3:** Antioxidant properties of T-CMC/SS dressings at different concentrations, investigated by DPPH and ABST radical scavenging assays and FRAP assay.

Dressing	DPPH Scavenging Activity (%)	ABTS Scavenging Activity (%)	FRAP Assay (mg Fe^2+^ Equivalents/mg Sample)
1%T-CMC/SS	3 ± 4	10.7 ± 0.4	1.52 ± 0.02
2%T-CMC/SS	10 ± 2	20 ± 2	2.1 ± 0.2
3%T-CMC/SS	13 ± 2	24 ± 1	3.7 ± 0.4

## Data Availability

The data presented in this study are available on request from the corresponding author.

## Supplementary Materials

The following supporting information can be downloaded at: https://www.mdpi.com/article/10.3390/polym15051090/s1, Figure S1: Release kinetics graphs: (a) Higuchi model and (b) Korsmeyer–Peppas model of 1%, 2%, 3%T-CMC/SS dressings; Figure S2: Representative photographs of inhibition zones of CMC, CMC/SS, 1%T-, 2%T-, and 3%T-CMC/SS dressings towards *E. coli* and *S. aureus*.
