# Supplementary material for: Turmeric Herb Extract-Incorporated Biopolymer Dressings with Beneficial Antibacterial, Antioxidant and Anti-Inflammatory Properties for Wound Healing
Source: Polymers (Basel). 2023 Feb 22;15(5):1090. doi: 10.3390/polym15051090 (PMC10007553; doi:10.3390/polym15051090)
Supplement: Supplementary file 1 [file polymers-15-01090-s001.zip › polymers-2221042-supplementary.pdf]

polymers

MDPI

Supplementary material

Turmeric Herb Extract-Incorporated Biopolymer Dressings with Beneficial Antibacterial, Antioxidant and Anti-Inflammatory Properties for Wound Healing

Piyachat Chuysinuan [1], Chalinan Pengsuk [2], Kriengsak Lirdprapamongkol [3], Thanyaluck Thanyacharoen [1], Supanna Techasakul [1], Jisnuson Svasti [3] and Patcharakamon Nooeaid [4,*]

[1] Laboratory of Organic Synthesis, Chulabhorn Research Institute, Bangkok 10210, Thailand
[2] Division of Biotechnology Technology and Agricultural Products, Faculty of Agricultural Product Innovation and Technology, Srinakharinwirot University, Ongkarak, Nakhon Nayok 26120, Thailand
[3] Laboratory of Biochemistry, Chulabhorn Research Institute, Bangkok 10210, Thailand
[4] Division of Polymer Materials Technology, Faculty of Agricultural Product Innovation and Technology, Srinakharinwirot University, Ongkarak, Nakhon Nayok 26120, Thailand
* Correspondence: patcharakamon@g.swu.ac.th; Tel.: +662-649-5000 (ext. 27167)

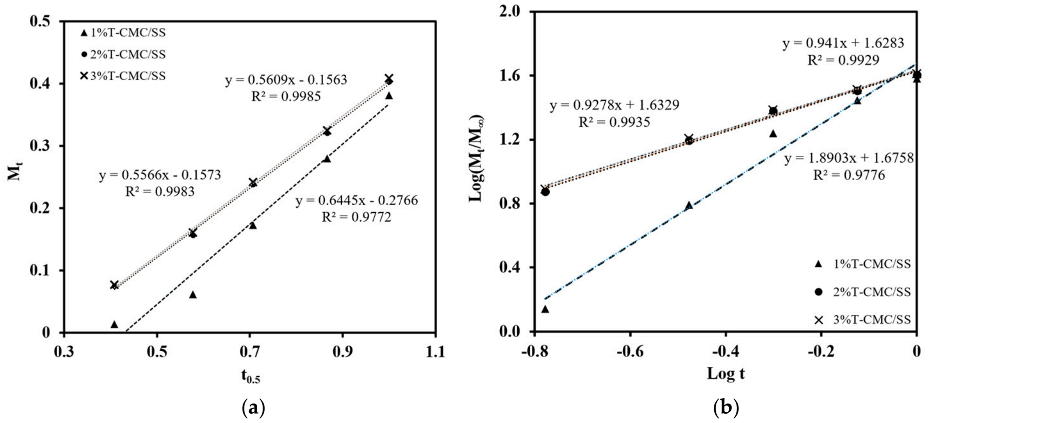

Figure S1. Release kinetics graphs: (a) Higuchi model and (b) Korsmeyer–Peppas model of 1%, 2%, 3%T-CMC/SS dressings.

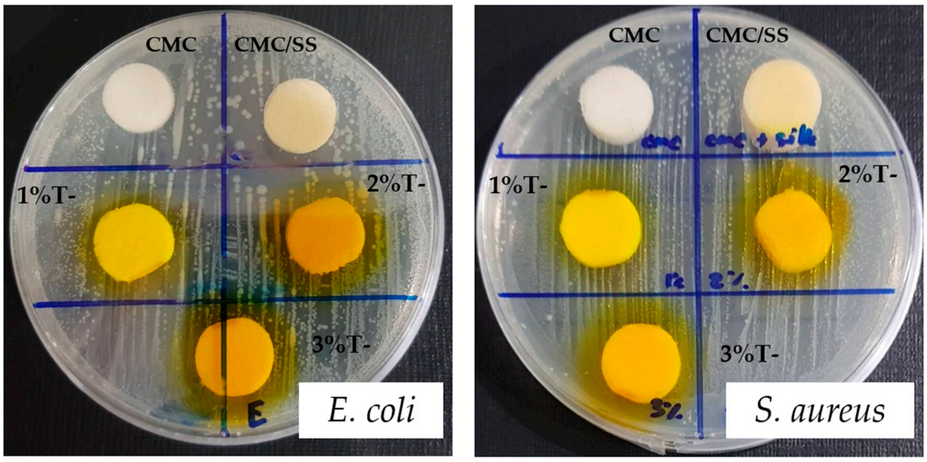

Figure S2. Representative photographs of inhibition zones of CMC, CMC/SS, 1%T-, 2%T-, and 3%T-CMC/SS dressings towards *E. coli* and *S. aureus*.
